# A Systematic Review of Unexplained Early Regression in Adolescents and Adults with Down Syndrome

**DOI:** 10.3390/brainsci11091197

**Published:** 2021-09-10

**Authors:** Madeleine Walpert, Shahid Zaman, Anthony Holland

**Affiliations:** Cambridge Intellectual and Developmental Disabilities Research Group, Department of Psychiatry, University of Cambridge, Cambridge CB2 8AH, UK; CIDDRG@medschl.cam.ac.uk (M.W.); shz10@medschl.cam.ac.uk (S.Z.)

**Keywords:** Down syndrome, early regression, idiopathic regression

## Abstract

A proportion of young people with Down syndrome (DS) experience unexplained regression that severely impacts on their daily lives. While this condition has been recognised by clinicians, there is a limited understanding of causation and an inconsistent approach to diagnosis and treatment. Varied symptomology and little knowledge of the cause of this regression have impacted on clinician’s ability to prevent or manage this condition. The purpose of this review was to examine the current evidence surrounding unexplained regression in adolescents and young adults, and to establish patterns that may be of use to clinicians, as well as raising awareness of this condition. Four areas were specifically reviewed, (1) terminology used to refer to this condition, (2) the symptoms reported, (3) potential trigger events and, (4) treatments and prognosis. A variety of terminology is used for this condition, which has constrained past attempts to identify patterns. An extensive number of symptoms were reported, however sleep impairment, loss of language and distinct changes in personality and behaviour, such as disinterest and withdrawal, were among the most frequently seen. Life events that were tentatively associated with the onset of a regressive period included a significant change in environmental circumstances or a transition, such as moving home or leaving school. Prognosis for this condition is relatively positive with the majority of individuals making at least a partial recovery. However, few patients were found to make a full recovery to their previous level of functioning and serious adverse effects could persist in those who have made a partial recovery. This is an under-researched condition with significant impacts on people with DS and their families. There are no established treatments for this condition and there is relatively little recognition in the research community. Further studies that focus on the prevention and treatment of this condition with controlled treatment trials are needed.

## 1. Introduction

Down syndrome (DS) is the most common syndrome associated with the presence of an intellectual disability, affecting approximately 3.3–6.7 per 10,000 individuals worldwide [1]. Family members and clinicians have noted the occurrence of cognitive deterioration and skills loss specifically in a small portion of adolescents and young adults, often without a distinct cause. This unexplained regression is profound and has a serious impact on both the individual and their families. A great number of different terms have been used in the diagnosis of this regression. Recently, “Down syndrome disintegrative disorder” (DSDD) and idiopathic regression in DS (IRDS) have been used more frequently. The latter term, IRDS, will be used in this review.

Despite the occurrence of IRDS being well-recognised by clinicians as affecting a minority of young people with DS, research in this area is sparse [2,3,4]. Characteristically, the symptoms and signs of this condition include significant impacts on the person’s cognitive and language functioning, their ability to perform daily tasks, a considerable loss of previously acquired daily skills, mild to severe alterations in personality and behaviour and the onset of social withdrawal. IRDS is described by families as having a profound effect on the abilities of the person with DS to live as they have previously been able to. This has a knock-on effect on members of their family and other carers, and often results in the need for major changes in their living situation and care needs.

This condition typically occurs in early adolescence to young adulthood and there are currently no confirmed causes or triggers and no consistent treatment pathways. In IRDS, presenting symptoms sometimes overlap with features of autism and dementia, however the age profiles for these other conditions are different. Autism spectrum disorder (ASD) presents in early childhood and a diagnosis of Alzheimer’s disease (AD), based on the onset of clinical symptoms, is most commonly made in the fifth decade. With dementia in people with DS, the neuropathological hallmarks are often seen earlier, (around the 40s) [5,6], but a clinical diagnosis of dementia is not usually made until the patient is in their 50s. This high risk of AD for people with DS is customarily theorised to be linked to the triplication of chromosome 21 and therefore the presence of three copies of the amyloid precursor protein gene, and the resultant lifelong overproduction of the beta-amyloid (Aβ) protein [7]. Despite the high risk of AD specific to people with DS, there is minimal evidence to suggest that AD presentation occurs at the age when individuals are most likely to be affected by IRDS. Furthermore, with IRDS there is often stabilisation and/or recovery of symptoms, as opposed to when it is dementia where progression with no recovery is what is to be expected. It is generally accepted that IRDS symptoms are not a consequence of either the above conditions and should be considered as separate.

A recent paper by Santoro et al. [4] reported the findings from a retrospective chart review of 35 people with DS and regression. Using a checklist of symptoms were classified into five core “features” including: (a) adaptive functions, (b) functional and procedural memory deficits (c) motor control impairment; (d) catatonia and; (e) disturbances associated with mental ill-health. The strengths of this study included the analysis of symptomatology in a group of people with DS, who experts had agreed had unexplained regression, and the use of an agreed checklist of symptoms. Most importantly, and uniquely in this field, this study compared symptomatology and test scores of patients with an aged-matched group of people with DS with no evidence of IRDS, thus helping to validate the recorded clinical observations. The authors do not report the temporal sequence of specific clinical symptoms. However, the majority of symptoms identified in those with IRDS were not experienced in the healthy controls, the exceptions to this being the mental health categorisations and externalising behaviours (hyperactive, irritable, disruptive, agitated) where there were no significant differences between IRDS and DS groups.

During the preparation of this systematic review another review paper was published summarising reported studies of regression in people with DS [8]. This paper identified language regression, mood disturbance and new onset insomnia as being particularly common features. They proposed that there were two potential causative mechanisms, one relating to immune dysfunction, and the other being stress related. Clinically, it was argued that an extensive work-up is still required to identify possible rare causes of regression, including the co-occurrence of other genetic disorders, such as Lesch Nyhan syndrome, in which a similar regression occurs but much earlier in life. Our systematic review complements this paper, drawing in greater depth on case studies as well as reports on case series of IRDS in adolescents and young adults with DS, and examining how symptoms cluster and co-present. Our primary objective, by extending the work undertaken by Rosso et al. [8], was to help improve diagnosis by heightening awareness of this condition among clinicians and providing further details of the main characteristics and their relationships to each other. We also reflect on terminology, clinical practice and possible causation.

Our review has focussed on observations from case studies and research on IRDS in adolescents and young adults with DS. The specific aims were to identify patterns of (a) symptomology, (b) potential trigger events and, (c) prognosis, treatments and outcomes. Possible causation will be considered to highlight the need for treatment trials for this condition based on the understanding of causal mechanisms. In addition to raising awareness of the condition we highlight the importance and necessity of further research of this condition.

## 2. Methodology

### 2.1. Identification of Articles

This systematic review was conducted according to the Preferred Reporting Items for Systematic Reviews and Meta-Analyses (PRISMA) statement [9] and was accepted to the Prospero platform (registration CRD42019156614). PubMed and Scopus databases were searched in November 2019 using the following search strategy: [“regression”] and [“Down* syndrome”]. Publication date and article language were not restricted; however, a filter was applied restricting articles to those involving human participants only. PubMed search fields included title and abstract, and in Scopus, title, abstract and keywords were included. As this systematic review did not contain independent research, ethical approval was not required, and consent procedures were not applicable.

### 2.2. Inclusion Criteria

Articles were examined for relevance by manually screening of the titles, abstracts and the keywords included. References of the retained articles were studied for further relevant papers, which were then examined against the same criteria.

Inclusion criteria were as follows:

- Research article involving at least one individual with DS.
- Age of patient under 35 years.
- Evidence of at least one regressive period that included changes to cognition, functioning and/or behaviour and personality.
- Regression identified did not progress to a clinical diagnosis of AD.

Cases with a co-diagnosis of pre-morbid autism in DS and early regression due to autism were not included. The inclusion of an upper age restriction was necessary in order to minimise the number of persons with DS whose symptoms may have been caused by the onset of AD. Furthermore, any individual cases included in papers who fell outside of this age limit were excluded from the review. Those with pre-existing features of catatonia were not excluded from this review due to the high incidence of presentation as part of IRDS. No other restrictions, such as language or year of publication, were included.

### 2.3. Data Collection Process

Data sought from the articles obtained included (a) terminology used to describe the condition (e.g., regression, catatonia), (b) symptomology (e.g., change in mental state, general mental functioning, level of living skills, sleep, appetite), (c) noted trigger events (e.g., life events such as bereavement, physical illness), (d) treatments prescribed (medication, psychological interventions, etc.) and (e) outcomes. For case studies this information was extracted on an individual basis and for cohort studies at a group level in accordance with the style of the individual article. Where possible this information was collated, otherwise two groups were considered for analysis (Group A—case study participants, Group B—cohort study participants).

## 3. Methodology

### 3.1. Article Search Results

**Figure 1:**
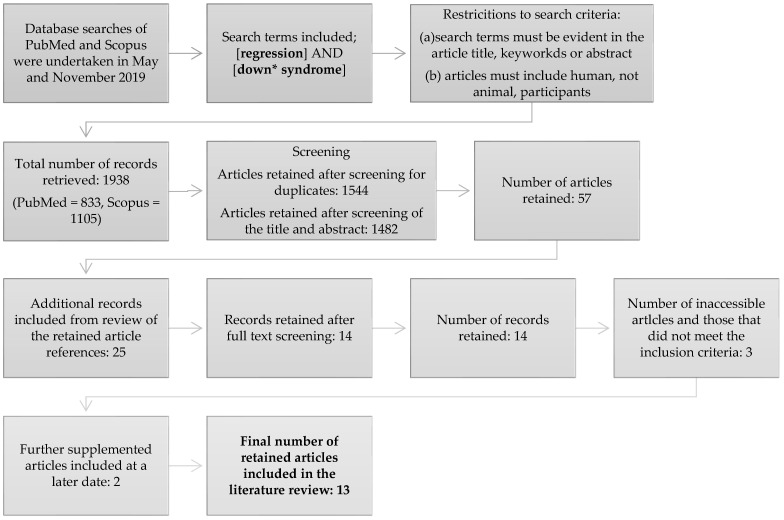
Conceptual framework showing article retrieval and inclusion process. Truncation was used in the search term Down* syndrome so as to capture alternate references such as Down’s and Downs syndrome.

A total of 1938 articles were identified from the initial search. Due to the search term “regression” without further specification, our search was deliberately over-inclusive. It was felt that due to the inconsistencies of terminology and labelling used in referring to this condition this was a necessary step in order to capture as many related articles as possible and to achieve our aim of collating the various terminologies used. Title, keyword and abstract review eliminated the vast majority of articles, leaving 57 articles for full-text review. A further four articles were sourced from references and full-text screening with the same inclusion criteria applied to these articles was completed. Ultimately 14 articles were retained. A full-text version could not be sourced for three articles, and two others were sourced at a later date, leaving a final total of 13 articles for inclusion in this review. The search pathway is shown in Figure 1 and the final included articles are summarised in Table 1.

### 3.2. Additional Comments and Exclusions

Articles where the age range of the participants extended outside of our 35-year upper age limit underwent an additional level of scrutiny. In research articles where cases could not be distinguished from each other, these were excluded, however in case studies or where this information was available, only the individual cases that did not meet our inclusion criteria were excluded from that particular review. Further exclusions included specified autistic regression and progression to AD. Two individual cases were excluded from this review based on the above criteria respectively. A ten-year-old female [12] and a 44-year-old male [10]. Table 1 reflects the number of participants after these additional exclusions. Case-control studies identified that did not distinguish between young children and adolescents could not be included in the review [4,21,22].

## 4. Results

### 4.1. Patient Demographics

The total number of people with DS included in the subsequent analyses was 186. Case report data were available for 39 patients with DS, these formed Group A. Group B represented the 147 patients without individual case study reports, the cohort group. One hundred patients (53.7%) were female and 86 (46.2%) male. The mean age of onset was 20.97 years. However, this statistic is not truly representative for two reasons. First, the cohort studies provided only mean age and age range, therefore individual ages could not be entered into this analysis. Secondly, differences between age of onset and age at presentation were not always specified in the case studies. In comparison to other studies that have looked at average age of onset, our estimate may be considered high; Santoro et al., [4] for example identified 17.5 years as the typical age that regressive symptoms first appear.

### 4.2. Descriptive Terminology

There were considerable problems with the search strategies used in this systematic review. One of which was deliberate. The use of the word “regression” in the search terms led to massive overlap with usage in statistical terminology. This led to many retrieved articles being unrelated to the review topic. It was however necessary to include this term in order to capture all relevant articles.

One of our primary intentions of this review was to evaluate the wide variety of terminologies that are used to describe and diagnose this condition (see Table 2). Analysis of the terms used across the 13 papers entered in this review revealed that five different terms including the word “regression” were used, as well as a further 10 different descriptors not including the word regression. These terminologies have been grouped and the frequency of their appearance is noted in Table 2. Multiple terms were often used in single papers, presumably reflecting the uncertainty and inconsistency in this area.

### 4.3. Symptom Severity and Diversity

**Figure 2:**
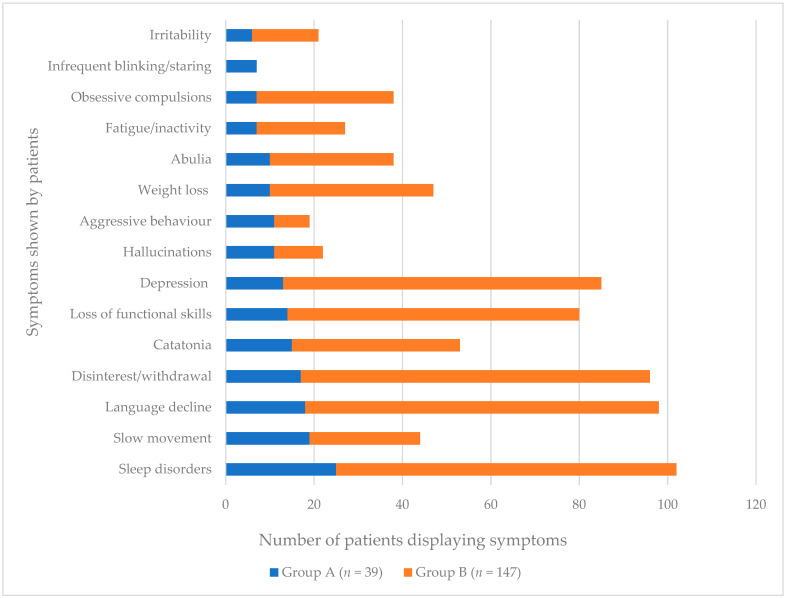
Number of cases in Group A (case study patients) and Group B (cohort study patients) displaying most prevalent symptoms of IRDS.

The majority of studies included in this review provided detailed information regarding patient symptoms. In our analysis we first assessed the qualitative data from the case studies (Group A). Our aim was to establish the most commonly reported symptoms from Group A, and then supplement these with the data from Group B. Additional symptoms that occurred in <10% of case studies reviewed were not included in this analysis or in Figure 2. Some Group B data could not be included as no symptoms were recorded [3]. In addition, symptoms were frequently reported differently between studies, for example, “catatonia” and “slowness of movement” were grouped as one category in one study [18], and “depression” and “compulsions” were grouped together in another [4]. To avoid missing the subtleties of these symptoms it was decided that, in these circumstances, patients should be recorded as having both symptoms.

From the results of this analysis, 15 independent symptoms were identified that were present in more than 10% of Group A patients. Some symptoms were reported heavily in individual studies and yet did not feature in others. For example, abulia was included in only two papers [4,12], but featured prominently in both. In Akahoshi et al. [12] 10 of the 12 of patients included were described as showing signs of abulia and in Santoro et al. [4] 28 of 35 patients were recorded as having a symptom under the heading of “motor control”, which included the features of abulia, avolition, and mutism.

Figure 2 shows the number of cases in each of the two groups of patients reported as having a particular symptom. Sleep disorders were the most commonly reported symptom in both groups and across all patients. Other highly occurring symptoms included language decline, disinterest/withdrawal, depression and loss of functional skills (self-care, toileting etc.). Onset or increase of previously present autistic characteristics were reported in three people with DS although some studies had excluded patients with co-morbid ASD [3,12]. Another symptom of particular note is weight loss, and in some cases the onset of what was described as anorexia nervosa was reported. Weight loss and poor appetite are not generally common in people with DS.

**Figure 3:**
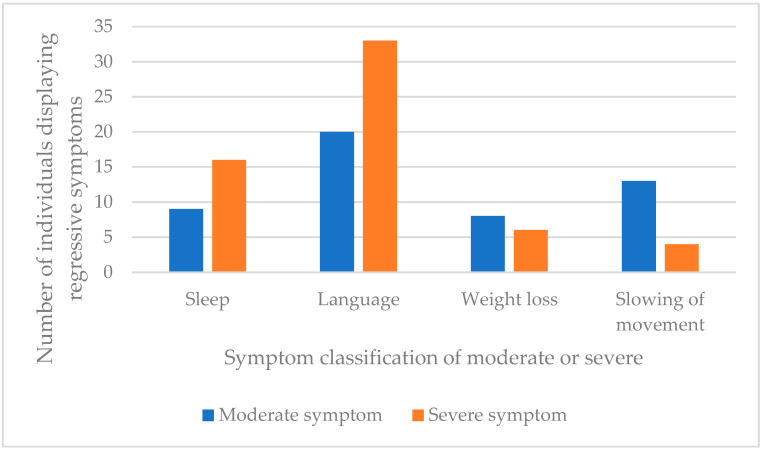
Number of case study patients (Group A, *n* = 39) where further details were given on the severity of the symptom.

With four of the symptoms listed in Figure 2 it was possible to determine the severity based on the vocabulary used to describe the symptoms. Reviewer determined categorisation of severity of symptoms was made based on descriptions given in the case studies, shown in Table 3. Figure 3 shows the prevalence of the moderate and severe symptoms identified in the case study data (Group A patients). It was not possible to complete this analysis in the cohort studies (Group B) due to the grouping of many of symptoms together which was not consistent across studies.

What is striking from this analysis is the number of patients considered to have developed severe impairments of their language skills, i.e., becoming mute or losing most of their previously acquired language abilities. Sleep impairment was also more likely to be severe than moderate.

A second area of analysis sought to identify patterns of symptom co-presentation in those people with DS in the case studies (Group A). Patterns identified for this are shown in Appendix B. Sleep disorder, deterioration in language, and becoming withdrawn and disinterested were the symptoms that, if present, were associated with the full range of symptomatology, rates of co-morbidity with other symptom clusters in most cases being above 50%. In contrast, obsessive compulsive behaviours, fatigue, and abnormal blinking and gaze are associated with co-morbidity rates with other symptoms of well under 50%. What cannot be determined is whether such observations are a manifestation of where the various patients were in the time course of the regression and/or whether this is a manifestation of the maximum level of severity overall. The fact that sleep disturbance is one of the reported early symptoms perhaps indicates that the early pathophysiology of IDRS involves the hypothalamus. However, such pathophysiology would need to extend beyond the hypothalamic and limbic systems to account for the onset of motor symptoms. Mapping the course of symptom development and ultimately recovery through a longitudinal study would provide valuable information, both in terms of clinical management but also inform as to the likely underlying pathophysiological course. This analysis was not possible for the cohort studies as there was no indication as to which participants expressed multiple symptoms. As outlined in Table 3, symptoms reported include both their severe and moderate forms.

### 4.4. Events Preceding Regression

**Figure 4:**
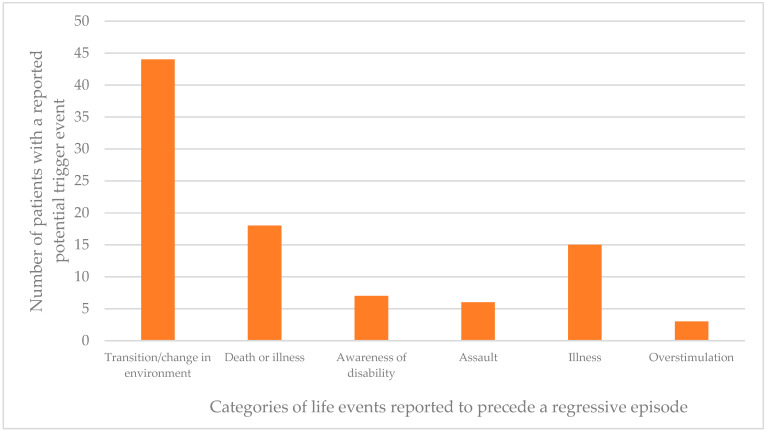
Number of cases with reported life events close to the time of a regressive episode (*n* = 93). Events included in this figure are those that occurred in more than one individual.

Records of life events occurring prior to the onset of regression were often referred to as “triggers” or “events” in the articles reviewed. The term “trigger” implies a causal effect for which we cannot be certain, in fact, it is likely that many more people with Down syndrome experience these same life events and do not develop IRDS. Among the articles reviewed this information was recorded for a total of 93 patients. Data were collected from the reviewed studies where reports of the same life event preceding a regressive episode was evident in >1 patient. Figure 4 shows the number of patients identified as experiencing such an event close to the time of their regressive episode onset.

**Figure 5:**
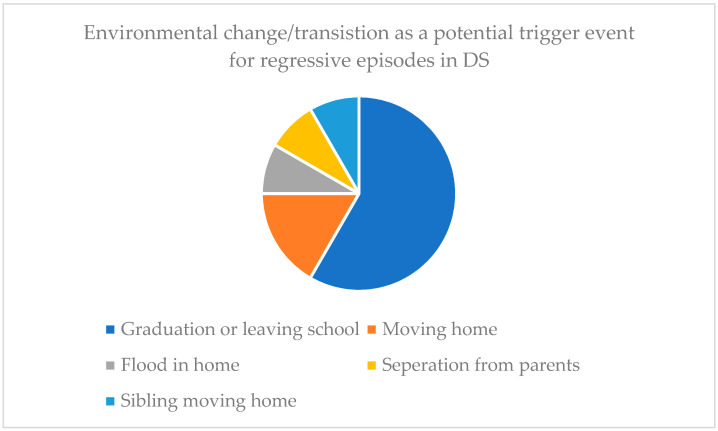
Breakdown of the environmental or transitional event circumstances that may have preceded a regressive episode (*n* = 12).

Figure 4 shows that “transition/change in environment” was the most commonly reported life event that occurred around the time of an individual regressive episode. Twelve of the 44 patients where this was suggested as a potential preceding event also included additional information regarding the circumstances. Seven cases suggested there was an association between graduation or leaving high school and the onset of a regressive period (see Figure 5).

### 4.5. Brain Abnormalities

Articles that provided brain-imaging data for the participants with DS were limited. The majority reported no abnormalities using brain MRI [15,17,19] or EEGs [15,19]. MRI data in one case study showed what were reported as senile changes in the five people with DS for whom they had imaging data, including ischemic changes in the cerebral white matter, hippocampal atrophy and basal ganglia calcification [12]. Mircher et al. [18] provided the most substantial brain imaging data, with records of 15 people with DS who underwent structural MRI. Eleven were reported as having normal brain structure, while the remaining four showed indications of abnormal brain structure, specifically, thin hippocampus (1), para hippocampal sulcus verticalisation (1), cerebellar hypotrophy (1) and cortical and cerebellar hypotrophy (1). Brain abnormalities were also reported using MRI neuroimaging in a single case study of a 19-year-old man with DS, however the nature of the abnormalities were not commented on further [15]. Of 11 EEGs conducted, all patients were reported as having normal EEG activity and from 23 polysomnography tests, two patients were reported as having abnormal findings, no further details were given. Additional brain abnormalities recorded included calcification of the pallidum, pineal body and habenular commissure, as well as low signal intensity in the pallidum and high signal intensity in the pyramidal tract and crossing of the superior cerebellar peduncles. Due to the absence of a comparison group or other studies to support these findings, it is not possible to determine from these results the significance with respect to IRDS.

### 4.6. Medications, Interventions and Outcomes

An important aim of this review was to give insight into the types of treatments and interventions used for this condition. As there are no treatment trials or controlled trials in this area, we have compiled a list of the medications and interventions reported across all the articles and the recorded outcome for the individual with DS. These details can be seen in Appendix A. In the absence of controlled trials, it is not possible to provide a review on the efficacy of a treatment, however it is interesting to note the wide variation of medications administered, the majority of which were given for different durations, at different dosages and alongside different additional medications. Thus, it is impossible to do any direct comparisons but it is important to be aware of the variety in the current interventions used.

**Figure 6:**
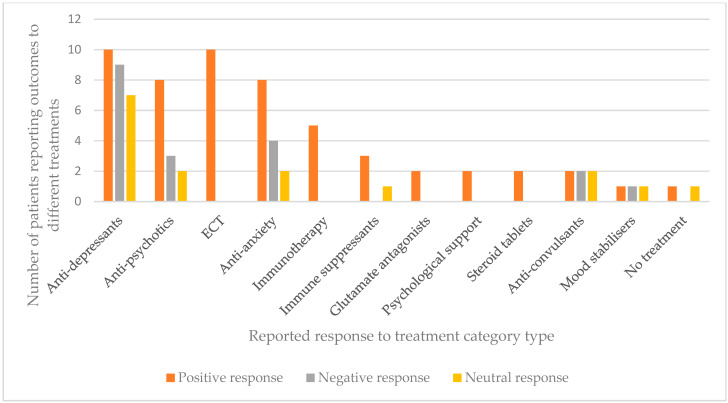
Bar chart showing the frequency of cases with administered drugs and treatments alongside outcomes for each type of treatment (*n* = 89). For more details on reported outcome see Appendix A.

Treatments that were administered to more than one person with DS have been recorded in terms of the response rate that was observed, either a positive, negative or no response (see Figure 6). It should be noted that in almost none of these examples were drugs/treatments the sole intervention, there was usually a combination of treatments that is not reflected in Figure 6; dosages and treatment lengths were also not recorded. For the purpose of this analysis, each treatment and response is entered as its own entity.

Anti-depressants and anti-psychotics were the most frequently used medications. Anti-depressants were reported as having had almost equal positive and negative effects with slightly fewer people with DS showing no changes after the medication. Of all the anti-depressants administered, clomipramine was the only one that was reported as resulting in improvement in all four people with DS it was given to, with one complete recovery. In this case 150mg clomipramine was administered once per day for six weeks. One hundred and fifty milligrams of desipramine had been administered previously but found to be ineffective. Electro-convulsive therapy (ECT) was found to be very effective. Of the 10 patients receiving this treatment four were reported to have made a full recovery [15,18], and a further four made significant recoveries to more than 80% of their baseline functioning [20]. Other patients showed complete resolution of some behaviours [19] and a “robust response” respectively [15]. There was, however, a high occurrence of discontinuation of ECT treatment, frequently resulting in relapse. Many patients receiving ECT had a preponderance of catatonia features [15,19,20], which may explain its effectiveness. Based on the wide variety of treatments employed and the large number that exhibited either no response or a negative response, clearly there is no consensus among practitioners regarding best treatment practice and there is a great need for improvement, both in our understanding of regression in people with DS and in the potential treatments.

### 4.7. Prognosis

**Figure 7:**
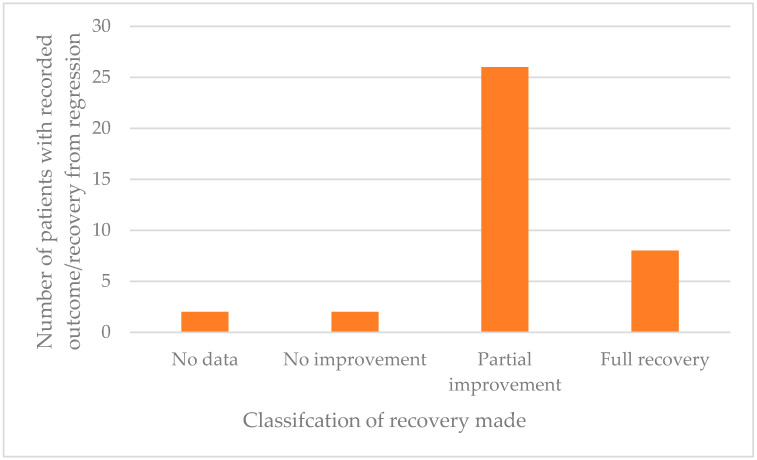
Recovery rate of 39 case study patients (Group A).

Overall, the majority of people with DS made some level of recovery from their regressive episode (Figure 7). Despite the range of different treatments used the majority appear, on the basis of clinicians’ reports, to have had mostly beneficial effects. Most intriguing is the finding that all patients treated with ECT showed a positive response. However, it is noteworthy and of concern that 66% of patients made only a partial recovery and did not return to their baseline functioning. Of those making a good recovery and even those returning to near pre-regression levels it was often reported that whilst most symptoms had been resolved, a specific behaviour or symptom remained. In some of these cases the remaining symptom(s) was very detrimental to daily life, such as persistent insomnia or unresolved mutism. Only eight people with DS (20%) made a full recovery to their baseline functioning, whilst two made no improvements and a further two withdrew from their treatment program and were not followed up.

## 5. Discussion

We undertook a systematic review to increase our understanding of the nature of early regression in adults and adolescents with DS. The standalone term “regression” was used for this systematic review in order to capture as many relevant articles as possible. Based on the overwhelming number of articles referencing only a form of statistical regression this is not a method that should be recommended. Out of 1938 articles identified in the search only a small number of independent articles including observational case and cohort studies with a total of 186 people with DS were identified. Within these articles, 39 people with DS were presented as case studies/vignettes, providing qualitative details about the nature of their regression, symptoms, treatments and prognosis. Contrary to previous observations reporting a clear preponderance of females [8], males represented just under half of the population identified in this review (46.2%). Previous reports have identified the impact of IRDS [23], however, there are many differing opinions on the interpretation and classification of IRDS, including the diagnoses of reactive depressive illness [14], and catatonia [15].

We identified the use of a total of 17 different descriptive terms to label this condition within the title, abstract and keywords in 13 articles. This review is by no means the first to notice the issues surrounding the description of this condition. Worley et al. [22] initiated the use of the term DSDD in their paper detailing autistic like regression in young children with DS. Of the articles included in this review that have been published since, few have continued with the use of DSDD [8,22]. However, other papers have referenced Worley et al., and repeated the concern of the lack of a unified term [16,18].

In this review, we grouped the symptoms that were noted within the cases studies (Group A) and those seen in the cohort studies (Group B). In the absence of a population-based study an estimate of the age-specific prevalence of IRDS was not possible. However, with the case study data (Group A) the description of symptoms was detailed and qualitative in nature thus allowing further insight into the severity of the symptoms. For the purpose of this review, observations concerning the 10 most predominant symptoms from Group A were identified, and then supplemented with data from Group B, the cohort studies. There were many individual cases of very specific changes and skill loss that were not necessarily representative of others with the condition, therefore symptoms noted in less than 10% of all cases were not reported in this analysis. Our priority was to identify the symptoms that were most commonly seen, the severity of those symptoms, and whether or not they were associated with the presence of other symptoms. Determining severity was especially problematic in this review as in the absence of baseline data it was difficult to quantify skill loss. In the case studies there was some additional information given about an individual’s prior behaviour and capabilities thus enabling some judgment about severity, however for consistency, the descriptive words used by the article were used to group symptoms.

In contrast to the Rosso et al. review [8], our review found that sleep disturbance was the most significant clinical feature. As in the other review, language skills decline and changes in behaviour, specifically becoming disinterested and withdrawn, were frequent. These latter symptoms were evident in around 50% of both Group A and B participants. For Group A we were able to cross-reference symptoms in an effort to establish patterns and high rates of symptom co-morbidity. We found that deterioration in sleep and speech was highly associated with almost all other symptoms, including slowness, weight loss and depression. Other associations that were seen less frequently included catatonia with skill loss, weight loss with increased slowness, and aggression with hallucinations.

Across the case studies that were reviewed there was a considerable difference in the type and amount of detail given. The majority of case studies reported in a narrative style without particular consistency in language or inclusion. This style provided the greatest detail and in-depth analysis. Studies where narratives were not used were slightly more problematic. For example, Akahoshi et al. [12] provided a table for their case reports with minimal qualitative data. It was considered that the subtleties of the symptoms may have been lost in an attempt to fit the criteria into pre-set categories. Eleven of the cases were described as showing signs of abulia (11) or hyperboulia (1), however, this symptom was not identified in any other case report narrative reviewed. Eight of these patients were also diagnosed with insomnia, the most severe of the sleep disorders reported and none with more moderate impairments. Whilst this may be a true reflection of the patients’ symptoms it is also possible that a reduced number of descriptors were decided on to fit pre-determined categories. The unique reporting methods were almost more in line with that of the cohort data. A limitation we are aware of is the impact that such results can have on review data, particularly in small samples such as these.

Severity of symptoms was deduced for participants (both Groups A and B) based on the language used to describe or classify the symptoms. It was clear that within the descriptions of sleep disorders and language decline, the presence of more severe symptoms significantly outweighed the more moderate symptoms. For these categories, this equates to more patients suffering from insomnia rather than disturbed sleep, and more patients described as experiencing mutism in comparison to less severe decline in language skills. Weight loss was also of interest as equal numbers of people with DS were reported as having suffered from loss of weight/appetite or a diagnosis of anorexia nervosa. Information was not given as to the exact nature of the symptoms that led to a diagnosis of anorexia nervosa, for example the nature and extent of body dissatisfaction, and it is likely that a diagnosis of anorexia nervosa in someone with DS may be difficult to make reliably. Interestingly, in the general DS population weight loss is not often reported. More typically the opposite, over-eating and weight gain is considered problematic, therefore, relatively high numbers of people with DS experiencing weight loss and receiving the diagnosis of anorexia nervosa may be an important indicator of symptomatology that is specific to this condition. The reasons behind appetite loss and weight change were not given in the articles reviewed, however, there is a strong co-presentation of disinterest and withdrawal in those whose weight changed, including four of those who were reported to have developed anorexia nervosa. Clinical features of IRDS must be identified if there are hopes of raising awareness of this condition, Table 4 presents the profile of IRDS.

On the surface, the statistics for recovery rate appear very positive. In our review only two individuals from Group A were reported as having made no improvement. However, 66% of patients made only a “partial recovery” (*n* = 26). This encompassed everything from slight improvement to near baseline functioning. Although making a recovery to “near baseline” may appear positive, in many cases one or more severe symptoms remained, including insomnia and mutism. In the case studies only eight people with DS recovered their full baseline abilities and functioning level after experiencing a regressive episode. The time course of IRDS remains uncertain in the absence of systematic longitudinal studies and it is also unknown whether there is a time point in the course of IRDS after which further recovery is unlikely.

Overall, the treatments used had varying results. Anti-depressants (including; clomipramine, bupropion, trazodone, fluvoxamine, desipramine, amitriptyline, nortriptyline and citalopram) were the most commonly administered drug type, the effects of which were almost equal between a positive, negative and no response. Similar numbers of positive and negative respondents were seen from the use of anti-anxiety drugs (mexazolam, bromazempam, benzodiazepines, lorazepam). Clomipramine exhibited a positive response in all people with DS it was given to, whilst ECT and immunotherapy appeared to have the most positive outcomes, with all patients exhibiting a positive response, although in each treatment the numbers were small (10 and 5 cases, respectively). Anti-psychotic medications (clozapine, levomepromazine, haloperidol, olanzapine, aripiprazole, ziprasidone and thiothixene) resulted in more positive responders than negative or no response. Of significant concern, both clinically and in terms of drawing any conclusions, is that in very few cases was a single treatment given independently and across the studies the length of administration and dosage varied, as were the other treatments that were alongside. For the purposes of this analysis, each intervention has been taken as an independent entity. Whilst this is far from ideal, it is impossible to accurately record response to a single treatment when multiple are prescribed in conjunction. There were no controlled treatment trials among the studies reviewed and in the absence of such trials it is impossible to be certain whether or not reported improvements following treatment are a manifestation of a treatment effect or just an indication of the natural history of IRDS in that individual.

Many of the symptoms presenting in IRDS are also seen in other conditions, such as depression and anxiety, or are seen in response to stress. These conditions may present atypically in people with DS [13], including the loss of functional skills, sleep impairment and reduced language [24]. In several studies, it was noted that people with DS were not experiencing depression prior to the episode, and many individuals were unresponsive to psychotropic drugs. Furthermore, some of the behaviours and changes, such as mutism, were never reversed despite making an otherwise complete recovery. This extent of persistent loss is not commonly seen in pure mood disorders.

A striking feature of IRDS is the age of onset, which is at an age when brain development is in its final stages and still susceptible to being disturbed. This age of risk for IRDS is later than that of classical autistic regression, or that observed in other rare neurodevelopmental disorders, and earlier than would be expected for dementia. Furthermore, the majority of studies have not reported abnormal imaging (MRI) or encephalographic (EEG) findings [15,17,19]. Other data showed senile changes in the five people with DS that received an MRI scan; however, there was no comparison or longitudinal data available [12]. Although routine medical screening (such as for thyroid disease, coeliac disease and vitamin D deficiency) undertaken in people with DS presenting with possible regression may be abnormal, it is unlikely such abnormalities are causative [4]. However, treatment of co-occurring medical conditions may improve prognosis.

The presentation of IRDS has been likened to that of dementia. Although the age of onset of dementia in people with DS is early compared to the general population it is still considerably later than the typical age of onset of IRDS. Myers and Pueschel [10] included a case study of a 44-year-old person with DS, excluded from this review due to our age inclusion criteria, who exhibited very similar symptoms to that of the younger participant, but the psychiatric diagnosis was AD. The major differences between the type of regression discussed in this review and dementia is the age of onset and, most crucially, that patients often show some recover from IRDS. No recovery is seen in those diagnosed with AD.

Many other diagnoses have been considered to explain this early regression, including psychotic illness, catatonia, mania, depression and anxiety. Autoimmune encephalopathy and mitochondrial dysfunction have been considered as possible underlying mechanisms [25,26]. Immune abnormalities are typical in children with DS [27]) and may be of aetiological significance. Interestingly one of the most effective treatments reported in this review were drugs impacting on the immune system, where all but one individual (who discontinued their use due to negative side effects) saw positive results, including one full recovery, and full recovery with the exception of persistent insomnia.

At present the underlying cause(s) of IRDS and why it appears to be specific to people with DS are unknown. One key question is whether we know enough about IRDS at present to argue that IRDS is a specific condition with a common, but as yet unknown, aetiology. Rosso et al. [8] argue that, as the cause(s) of IRDS is unknown, a full clinical work up in all cases of young people with DS who report such changes is required to identify possible explanatory and potentially different diagnoses. If, on the other hand, all clinically diagnosed cases of IRDS are in fact considered to have a common cause a further question is whether that is best explained as a consequence of the atypical presentation of a known co-morbid condition (e.g., catatonia), which occurs in other populations but happens to be more common in people with DS; or whether IRDS is a condition that has an aetiology that is unique and specific to DS, and ultimately can be linked back to the presence of trisomy 21. The paper by Miles et al. [20] interestingly reported on seven patients with DS who had developed regression and the authors proposed that in each case this regression was due to catatonia. Using the Bush–Francis Catatonia Rating Scale and demonstrating a positive response to intravenous lorazepam they argued that this was true catatonia. They also reported a good response to ECT and other treatments although recovery was often not maintained. Such observations would suggest that regression is due to a condition (in this case catatonia) that can affect anyone, but that people with DS at a specific age are particularly vulnerable. However, although people with other neurodevelopmental syndromes may have syndrome specific neuropsychiatric risks, to our knowledge similar episodes of regression in the same age range are rare in people with other neurodevelopmental syndromes. This is more in support of the hypothesis that IRDS is a DS specific condition. An exception to this is cases of regression in people with SHANK3 mutations (Phelan–McDermid syndrome), reported in two papers [28,29]. For people with Prader-Willi Syndrome, particularly those with the maternal uniparental disomy form, a regressive type of clinical deterioration can be seen in the same age group as IRDS but the symptomatology is much more obviously that of a psychotic illness [30]. Although there are some differences in symptom prevalence [8] the symptomatology that is described in both reviews would appear to separate IRDS from other potential aetiologies, such as affective disorder. However, such uncertainties as those described above are clearly hindering a rational approach to treatment development.

It is unknown whether there are specific risk markers and biomarkers that are either an indicator of vulnerability to IRDS or can map to the presence and severity of the IRDS and are potential indicators for underlying causative mechanisms. These need to be studied and markers identified and then followed over time. Whether there is a relationship between the risk for regression and the later risk of dementia is unknown and specifically whether there are genetic markers that affect the vulnerability to dementia (e.g., ApoE genotype and soluble TREM-2) are also associated with regression. And whether biomarkers of other potential mechanisms (e.g., myelodysplasia, leukopenia, macrocytosis), and inflammatory markers (e.g., inflammation related factors, cytokines) are important and need further investigation. Many of these markers have been identified as being associated with major psychiatric disorders.

A striking observation is the potential role of transitional life events and changes in environment as a potential trigger for regression. IRDS may be best considered as a condition triggered by stress and occurring in people with DS who have some additional genetic or acquired vulnerability (low resilience). Alternatively, IRDS is due to the occurrence of an acquired condition that results in a direct, and initially adaptive response in the brain to the insult and subsequently in a temporary and adverse effect on brain function. For example, an acquired insult may lead to the development of an inflammatory response in the brain resulting in an encephalopathy that subsequently completely or partially resolves.

### Limitations

There are several limitations with this review article that have mostly been discussed in the course of this review. First, the search strategy used to capture relevant articles vastly over-included studies and returned a huge number of results that were irrelevant. The difficulty with this search centred on the labelling words used to describe this condition, and that there is not a unified term. Our compromise, after trialling many search strategies, was to include the word “regression” without additional restrictions. While this returned the best results in so far as the few relevant articles, there were also a plethora of studies referencing only statistical regression that needed to be manually eliminated. It is recognised that it is not good practice to have such a large difference between the number of articles sourced and those retained, it was deemed necessary based on the confusion seen in the terminology used for this condition. Although the methodology was time consuming and over-inclusive, using the particular search criteria selected we are confident that the correct papers have been identified and included.

The articles included in this paper were all observational studies, both case and cohort studies. The positive aspects of observational data are that records are usually qualitative and highly descriptive, allowing for cross-referencing between symptoms to be explored and person-by-person outcomes evaluated based on interventions. The negatives are that the quality of evidence is low. Cochrane’s levels of evidence quality [31] describe randomised controlled trials as giving the highest quality of evidence and observational studies amongst the lowest. As such any conclusions drawn from the studies must be very cautiously considered. It is our hope that further insight and raising awareness will lead to a greater interest in research of this condition and promote controlled trials in the future.

Despite these limitations this review has provided insight into an under-researched condition with significant impacts on people with DS and their families. This review intends to bring to light a serious condition affecting a minority of young adults and adolescents, many of whom never recover their baseline functioning. It is important that we now seek to focus on prevention and treatment of this condition.

## Figures and Tables

**Table 1:** Summary of articles included in the systematic review.

Author	Number of Case Study Patients (Group A)	Number of Cohort Study Patients (Group B)	Gender (Female: Male)	Age
Myers and Pueschel (1995) [10]	8	8	4:4	Range 21–44 years
Capone, Aidikoff and Goyal (2011) [11]	0	33	14:19	Range 13–35 years Mean 22 years
Akahoshi et al. (2012) [12]	12	12	6:6	Range 13–29 years
Stein et al. (2013) [13]	1	1	Female	13 years
Capone et al. (2013) [14]	0	28	14:14	Male mean 21.8 years Female mean 20.3 years
Dykens et al. (2015) [3]	1	49	49% male	Range 13–29 years
Ghaziuddin, Nassiri and Miles (2015) [15]	4	4	2:2	Range 14–18 years
Jacobs et al. (2016) [16]	1	1	Male	19 years
Tamasaki et al. (2016) [17]	1	1	Male	15 years
Mircher et al. (2017) [18]	0	30	20:10	Range 12–30 years
Cardinale et al. (2018) [19]	4	4	3:1	Range 17–25 years
Santoro et al. (2019) [4]	0	35	53% female	9–34 years
Miles et al. (2020) [20]	7	0	6:1	18–33 years

**Table 2:** List of the terminology used to describe the group of patients within the article. Articles may have referenced multiple terminologies.

Regression Related Terminology	Times Used	Disorder Related Terminology	Times Used	Function Related Terminology	Times Used
Regression	2	Psychiatric disorders	1	Deterioration	1
Developmental regression	1	Down syndrome disintegrative Disorder	2	Clinical deterioration	1
Cognitive regression	1	New-onset mood disorder	1	Functional decline	1
Unexplained regression	2	Acute neuropsychiatric disorders	1		
Rapid regression	2	Depression/major depression	3		
Acute regression	2				
Total	10	Total	8	Total	3

**Table 3:** Descriptive terminology used in articles reviewed. Four of the symptoms identified from the case study data (Group A patients) were able to be analysed. Reviewer determined categorisation of “moderate” or “severe” impact.

Symptom	Moderate Symptoms	Severe Symptoms
Sleep	Restless sleep Poor sleep Disturbed sleep	Insomnia
Language	Vocal stereotypies Language decline Incoherent speech	Mutism
Weight loss	Weight loss Appetite loss	Anorexia nervosa
Slowing of movement	Slowness Slow movement	Immobility Becoming bedridden

**Table 4:** Proposed clinical features of IRDS.

Core Symptoms and Signs	Potential Triggers for Regression	Exclusions
New onset poor sleep	Transitions	Autism spectrum disorder presents in 5 years and above
Change in language output	(e.g., changes in an individual’s home/school/college routine)	Medical causes (incl. thyroid dysfunction and other conditions with autoimmune aetiology)
Abulia, withdrawal, disinterest, personality changes	Life events	New onset sensory impairment
Mood changes, loss of appetite and weight loss	Stressors	Age-related decrease in activity
Motor features–catatonia, stereotypies, extra-pyramidal signs		Other mental illness (e.g., depression)
Loss of skills (adaptive functioning)		Unlikely over the age of 40 years (dementia is possible)

## Data Availability

Not applicable.

## Appendix A

**Table A1:** Table showing the range of treatments administered across all papers on an individual basis alongside reported outcome.

Treatment/Intervention	Administered *n*	Positive Response	Negative Response	No Response
SSRI—fluvoxamine	5	3 “Symptoms improved” “Moderate improvement” “Significantly better”	0	2 “No improvement” “No change”
Amantadine	2	2 “Partial improvement” “Partial improvement”		
Levomepromazine	2	2 “Partial improvement” “Partial improvement”		
Haloperidol	4	3 “Improvement” “Partially improved” “Improvement for 6 months”		1 “Unsuccessful”
Mexazolam	1	1 “Improvement”		
Bromazepam	1	1 “Partial improvement”		
Carbamazepine	2	1 “Partial improvement”		1 “Unsuccessful”
Clomipramine	4	3 “Partial improvement” “Partial improvement” “Complete recovery” “Slight improvement, side effects”		
Romethazine	1	1 “Partial improvement”		
Lorazepam	10	4 “Partial improvement” “80% return to baseline” “Responded to” “Showed increase” “Significant improvement”	1 “Negative effects”	5 “No consistent improvement” “No consistent improvement” “No consistent improvement” “No change” “Ineffective”
Methylprednisolon	3	3 “Dramatic improvement” “Immediate improvement” “Many behaviours resolved”		
IVIG	4	4 “Full recovery” “Steady improvement” “Lots of symptoms resolved” “Resolution of everything except insomnia”		
Mycephenolate	1			1 Discontinued no data
Oral steroid	2	2 “Immediate improvement” “Lots of resolved symptoms”		
Rituximab	1	1 “Improvement”		
Electro-convulsive therapy	10	10 “Some behaviours completely resolved” “Complete recovery” “Complete recovery” “Complete recovery” “Complete recovery” “Robust response” “Significant improvement” “Return to almost baseline” “Excellent response” Strong response”		
Benzodiazepines	1	1 “Partial improvement”		
Anti depressants	1	1 “Steady return to baseline”		
Positive airway pressure for obstructive sleep apnoea	1	1 “Steady return to baseline”		
Psychological support	2	2 “Steady return to baseline” “Moderate improvement for 6 months”		
Donepezil	1	1 “Return to baseline”		
Acetylcholinesterase inhibitor	1	1 “Return to baseline”		
Bupropion	1		1 “Worsening of catatonia and further decline”	
Trazodone	4	1 “Some improvement”	2 “Worsening of catatonia and further decline” “Worsening and decline”	1 “Unsuccessful”
Olanzapine	1		1 “Worsening of catatonia and further decline”	
Aripiprazole	1		1 “Worsening of catatonia and further decline”	
Ziprasidone	1		1 “Worsening of catatonia and further decline”	
Lithium	3	1 “Improvement”	1 “Worsening of catatonia and further decline”	1 “No improvement”
Clozapine	1	1 “85% return to baseline”		
Desipramine	6	1 “Moderate improvement for 6 months”	1 “Worsening of symptoms”	4 “No change” “No effect” “Unsuccessful” “Unsuccessful”
Thiothixine	2	1 “Complete recovery”		1 “No change”
Amitriptyline	1	1 “Complete recovery”		
Nortriptyline	1			1 “Unsuccessful”
Clonazepam	2	1 “Partial improvement”		1 “Unsuccessful”
Ethosuximide	1			1 “No effect”
Lidexamfetamine	1		1 “Worsening”	
SSRI citalopram	1		1 “Worsening”	
Amiloride	1	1 “Improvement”		
Lamotrigine	1			1 “No effect”
Antipsychotic treatment	1	1 “Good response”		

Note: dosages and additional medications/treatments are not reported.

## Appendix B

**Table A2:** Table showing co-presentation of symptoms in Group A patients.

	Sleep (*n* = 22)	%	Language (*n* = 14)	%	Withdrawal and disinterest (*n* = 14)	%	Slowness and immobility (*n* = 14)	%	Weight loss and anorexia (*n* = 12)	%	Depression (*n* = 11)	%	Hallucinations (*n* = 11)	%	Abulia (*n* = 10)	%	Skill loss (*n* = 10)	%	Catatonia (*n* = 8)	%	Aggression (*n* = 7)	%	Irritability (*n* = 6)	%	Obsessive compulsions (*n* = 6)	%	Fatigue (*n* = 5)	%	Abnormal blinking and gaze (*n* = 4)	%
Sleep	0		10	71	9	64	8	57	10	83	9	82	7	64	7	70	6	60	6	75	6	86	2	33	5	83	4	80	2	50
Language	10	45	0		3	21	7	50	4	33	7	64	6	55	2	20	6	60	6	75	4	57	2	33	1	17	0	0	2	50
Withdrawal and disinterest	9	41	3	21	0		6	43	5	42	5	45	4	36	8	80	2	20	0	0	2	29	3	50	4	67	3	60	0	0
Slowness and immobility	8	36	7	50	6	43	0		7	58	5	45	4	36	2	20	4	40	5	63	2	29	1	17	3	50	2	40	4	100
Weight loss and anorexia	10	45	4	29	5	36	7	50	0		3	3	4	36	2	20	2	20	2	25	2	29	1	17	1	17	4	80	2	50
Depression	9	41	7	50	5	36	5	36	3	25	0		4	36	0	0	2	20	6	75	2	29	1	17	0	0	0	0	2	50
Hallucinations	7	32	6	43	4	29	4	29	4	33	4	36	0		0	0	4	40	0	0	2	29	0	0	0	0	0	0	3	75
Abulia	7	32	2	14	8	57	2	14	2	17	4	36	0	0	0		0	0	2	25	2	29	0	0	0	0	0	0	3	75
Skill loss	6	27	6	43	2	14	4	29	2	17	2	18	6	55	2	20	0		2	25	0	0	1	17	0	0	2	40	0	0
Catatonia	6	27	6	43	0	0	5	36	2	17	2	18	0	0	2	20	6	60	0		0	0	0	0	0	0	3	60	0	0
Aggression	6	27	4	29	2	14	2	14	2	17	2	18	2	18	2	20	2	20	0	0	0		2	33	0	0	1	20	0	0
Irritability	2	9	2	14	3	21	1	7	1	8	1	9	0	0	3	30	1	10	2	25	2	29	0		2	33	0	0	2	50
Obsessive compulsions	5	23	1	7	4	29	3	21	1	8	0	0	0	0	4	40	0	0	0	0	4	57	2	33	0		0	0	0	0
Fatigue	4	18	0	0	3	21	2	14	4	33	0	0	0	0	0	0	0	0	0	0	2	29	2	33	0	0	0		1	25
Abnormal blinking and gaze	2	9	2	14	0	0	4	29	2	17	2	18	3	27	1	10	2	20	1	13	0	0	0	0	0	0	1	20	0	
